# Therapeutic efficacy of artemether-lumefantrine in the treatment of uncomplicated *Plasmodium falciparum* malaria in Chewaka District, Ethiopia

**DOI:** 10.1186/s12936-020-03307-4

**Published:** 2020-07-10

**Authors:** Abdulhakim Abamecha, Daniel Yilma, Wondimagegn Addisu, Hassan El-Abid, Achim Ibenthal, Harald Noedl, Delenasaw Yewhalaw, Mohieddine Moumni, Alemseged Abdissa

**Affiliations:** 1 School of Medical Laboratory Science, Institute of Health, Jimma University, Jimma, Ethiopia; 2 Department of Internal Medicine, Institute of Health, Jimma University, Jimma, Ethiopia; 3 Biotechnology and Bio-Resources Development Laboratory, Faculty of Sciences, Moulay Ismail University, Meknes, Morocco; 4 Faculty of Science and Art, HAWK University, Gottingen, Germany; 5 Malaria Research Initiative Bandarban (MARIB), Vienna, Austria; 6 Armauer Hansen Research Institute, Addis Ababa, Ethiopia; 7 Department of Biomedical, College of Public Health and Medical Science, Mettu University, Mettu, Ethiopia; 8 Tropical and Infectious Diseases Research Center (TIDRC), Jimma University, Jimma, Ethiopia

**Keywords:** Therapeutic efficacy, Artemether-lumefantrine, Uncomplicated malaria, Ethiopia

## Abstract

**Background:**

The efficacy of artemether-lumefantrine (AL) for treatment of uncomplicated *Plasmodium falciparum* malaria in south-western Ethiopia is poorly documented. Regular monitoring of drug efficacy is an important tool for supporting national treatment policies and practice. This study investigated the therapeutic efficacy of AL for the treatment of *P. falciparum* malaria in Ethiopia.

**Methods:**

The study was a one-arm, prospective, evaluation of the clinical and parasitological, responses to directly observed treatment with AL among participants 6 months and older with uncomplicated *P. falciparum* malaria. Real-time polymerase chain reaction (PCR) and nested PCR reaction methods were used to quantify and genotype *P. falciparum*. A modified protocol based on the World Health Organization 2009 recommendations for the surveillance of anti-malarial drug efficacy was used for the study with primary outcomes, clinical and parasitological cure rates at day-28. Secondary outcomes assessed included patterns of fever and parasite clearance. Cure rate on day-28 was assessed by intention to treat (ITT) and per protocol (PP) analysis. Parasite genotyping was also performed at baseline and at the time of recurrence of parasitaemia to differentiate between recrudescence and new infection.

**Results:**

Of the 80 study participants enrolled, 75 completed the follow-up at day-28 with ACPR. For per protocol (PP) analysis, PCR-uncorrected and-corrected cure rate of AL among the study participants was 94.7% (95% CI 87.1–98.5) and 96% (95% CI 88.8–99.2), respectively. For intention to treat (ITT) analysis, the cure rate was 90% (95% CI 88.8–99.2). Based on Kaplan–Meier survival estimate, the cumulative incidence of failure rate of AL was 3.8% (95% CI 1.3–11.4). Only three participants 3.8% (95% CI 0.8–10.6) of the 80 enrolled participants were found to be positive on day-3. The day three-positive participants were followed up to day 28 and did not correspond to treatment failures observed during follow-up. Only 7.5% (6/80) of the participants were gametocyte-positive on enrollment and gametocytaemia was absent on day-2 following treatment with AL.

**Conclusions:**

The therapeutic efficacy of AL is considerably high (above 90%). AL remained highly efficacious in the treatment of uncomplicated malaria in the study area resulted in rapid fever and parasite clearance as well as low gametocyte carriage rates despite the use of this combination for more than 15 years.

## Background

Malaria, caused by infection with *Plasmodium* protozoan parasites, threatens over half the world’s population [1]. Despite concerted efforts, which have considerably reduced the burden of mortality and morbidity in recent years, malaria remains a major public health threat [2, 3]. In 2017, over 219 million cases and 435,000 deaths were reported [2]. Approximately 92% of the cases and 93% of the deaths were from sub-Saharan Africa [2]. Between 2000 and 2015, the widespread adoption of artemisinin-based combination therapy (ACT), the increased use of insecticide-treated nets (ITNs) and indoor residual spraying (IRS) against the *Anopheles* mosquito vector, decreased the global number of malaria deaths by an estimated 37% [4]. Recently, these fragile gains are, jeopardized by the emergence and spread of drug resistance in the parasite and insecticide resistance in the mosquito vector [5].

Ethiopia is also one of the many malaria epidemic-prone countries in Africa [6]. The trends in malaria over the past five years have also shown a decline in malaria cases and reduced epidemics [7]. In 2014/2015, Ethiopia reported 2,174,707 malaria cases and 662 reported malaria deaths among all age groups which is 98% reduction compared to 41,000 estimated deaths in 2006 [7, 8]. The key interventions which have been contributing to such significant decline includes: introduction of prompt and effective treatment with artemisinin-based combinations to treat uncomplicated *Plasmodium falciparum* malaria, the distribution of long-lasting insecticidal nets (LLINs), indoor residual spraying (IRS); and to a lesser extent environmental management [7–9]. Following this, Ethiopia has also set a goal to eliminate the disease by 2030 [10, 11].

ACT is the first-line treatment for uncomplicated *P. falciparum* malaria and has been instrumental in reducing malaria burden [12, 13]. As yet the majority of endemic prone areas have little or no drug resistance to ACT, and its high efficacy (~ 95%) at clearing parasitaemia has been extensively demonstrated from clinical trials [14, 15]. However, there is limited information on ACT effectiveness in routine health care when treatment is not monitored.

Resistance of *Plasmodium* species to artemisinin has been reported from eastern and southern Asian countries which threatens malaria control and elimination efforts worldwide [16–18]. For the purpose of ensuring good performance and detection of emergence of resistance of anti-malarial drugs, especially those used as a first-line and second-line treatment in a country, the World Health Organization (WHO) recommends regular monitoring of their efficacy at least every two years in malaria-endemic countries [19].

Early diagnosis and prompt treatment is one of the main strategies in malaria prevention and control and it is also the key to reducing morbidity and preventing mortality in Ethiopia [6]. According to the President’s malaria initiative Ethiopia malaria operational plan fiscal year 2018, 60–70% of the total projected numbers of malaria cases are due to *P. falciparum* and to be treated with AL from year 2017–2019 [7]. The emergence and spread of both artemisinin and partner drug resistance threatens the efficacy of ACT and subsequently undermines the treatment of uncomplicated falciparum malaria, which is to eliminate all parasites from the body and prevent progression to severe disease [12, 21]. It is, therefore, necessary to generate continuous data on the therapeutic efficacy of first-line ACT to ensure real-time evidence-based review of national treatment policies as and when necessary. Since the introduction of ACT in Ethiopia in 2004, there have been few studies on therapeutic efficacy of AL [22–24]. This paper presents data on the therapeutic efficacy of AL for the treatment of uncomplicated falciparum malaria, the prevalence of day three parasitaemia, which has previously been used as a surrogate for artemisinin (partial) resistance and patterns of fever and parasite clearance.

## Methods

### Study setting and period

The study was conducted in Ilu Harar Health Centre, Chewka district, Buno Bedele Zone, Southwest Ethiopia during September–December 2017. Chewaka district is located in Buno Bedele zone, Oromia regional state about 570 km southwest of Addis Ababa. The district has 26 administrative kebeles (villages) and has an altitude ranging from 1600 to 2000 above sea levels. As in most other areas of the country, malaria transmission in Chewaka follows rainy seasons, with transmission peaking in the months between September and December and between April and May. The main malaria control strategies in the district includes: IRS, LLINs and malaria case management using ACT [6].

### Study design and participants

This was a prospective study of the clinical and parasitological efficacy of AL to directly observed therapy for uncomplicated *P. falciparum* malaria according to WHO revised protocol for malaria drug therapeutic efficacy study [20].

#### Inclusion criteria

Febrile patients (axillary temperature ≥ 37.5 °C or having history of fever within the previous 24 h, who fulfilled WHO revised protocol for malaria drug therapeutic efficacy study [20] and signed an informed consent were included in the study. Briefly, all participants over 6 months of age and body weight > 5 kg, and microscopically confirmed *P. falciparum* mono-infection with asexual parasitaemia of 1000–100,000 parasites/μl of blood, non-pregnant or non-breast-feeding women, permanently living within the health Centre catchment area (5–10 km radius) during the study period were recruited.

#### Exclusion criteria

Evidence of mixed of mixed or mono-infection with *Plasmodium* species other than *P. falciparum*, haemoglobin (Hb) level ≤ 5.0 g/dl, AL intake within the previous 2 weeks, inability to take oral medication or continuous vomiting, known hypersensitivity to AL, severe malaria or other danger signs, severe malnutrition, febrile conditions due to diseases other than malaria (e.g. measles, acute lower respiratory tract infection, severe diarrhoea with dehydration) or other known underlying chronic or severe diseases (e.g. cardiac, renal and hepatic diseases, human immunodeficiency virus (HIV)/acquired immunodeficiency syndrome (AIDS) cases); and regular medication which may interfere with AL pharmacokinetics.

### Treatment and follow-up

AL [Batch: DYI476065; Mfg: 03, 2016; Exp: 02, 2018] which was manufactured by Ipca Laboratories Ltd [Plot №: 255/1; Athal, Silvassa 396 230 (D & NH), India] was provided by the Ethiopian FMoH through WHO support. Drug dosage was determined according to the revised WHO weight-based guideline [12]. Briefly, participants were treated with the standard six-dose regimen of AL given twice daily for three consecutive days under direct observation of a study nurse/public health officer and administered with a milk biscuit to ensure good absorption. The participants were then observed for 30 min to ascertain retention of the drug. Participants who vomited during the observation period were re-dosed with the same drug and observed for an additional 30 min. Participants with repeated vomiting were withdrawn from the study and treated as severe malaria according to national standard treatment guidelines [6].

On day 0 (enrollment day), participants who were successfully treated with the first dose of AL were given an appointment card bearing patient name and identification code and next scheduled visit date, and the evening dose to be administered at home by health extension workers. Participants were then advised to come back for treatment the next 2 days (day-1, day-2). Scheduled follow-up visits were on day-3, day-7, day-14, day-21, and day-28. There were unscheduled visits as well when a participant felt sick. Microscopy was performed during each subsequent visit to determine infection status, species, and parasite density.

### Haemoglobin measurement

Finger-pick blood samples were used to measure haemoglobin using a portable spectrophotometer (Haemocue).

### Parasitological assessment

#### Microscopic analysis

Thick and thin blood films were collected from each patient at screening. Blood films were also obtained on days 1, 2, 3, 7, 14, 21, 28 and any other day, if the patient returned due to some complaints spontaneously [20]. Giemsa working solution with buffering PH of 7.2 was used to stain the smears. Double-slide blood smears were prepared; one stained rapidly with 10% Giemsa for 10–15 min to screen for recruitment, and the next stained with standard 3% Giemsa for 30–45 min as recommended elsewhere [20]. The blood smears were examined by two microscopists blinded to each other results at a magnification of 1000 × to examine parasite positivity, to identify parasite species and determine parasite density. Asexual and sexual stage of the parasite was determined from Giemsa-stained thick blood smears and enumerated against the number of parasites per 200 white blood cells on day 0, based on an assumed density of 8000 white blood cells per μl of blood. A blood smear was declared negative after examination of 1000 white blood cells [25, 26]. Discrepant results in terms of parasite positivity, species or density (by > 25%), a third blinded, independent microscopist re-examined the blood slides. For parasite species and positivity, two concordant results were considered the final result, while for parasite density, the average of the two closest estimates of parasitaemia was considered final.

### Molecular analysis

#### Screening of Plasmodium genus with qPCR

Primerdesign™ Genesig standard kit for *Plasmodium* spp. (all species) genomes was assayed for the in vitro quantification of all *Plasmodium* species genomes by targeting the 18S ribosomal RNA (18S) gene according to the protocol of Primerdesign™ Ltd [27]. Each reaction was performed in duplicate and the cycle threshold number (Ct) was determined as their mean. A sample was considered positive if the fluorescent signal was detected in at least one replicate; conversely, if no signal was detected within 40 cycles, a reaction was considered negative.

#### Species-specific qPCR

*Plasmodium falciparum* genome was analysed for the in vitro quantification of *P. falciparum* genomes by targeting the *plasmepsin 4* gene according to the protocol of Primerdesign™ Ltd [28].

#### Molecular genotyping of msp-1 and msp-2

Dried blood spots were obtained for PCR analysis at enrolment (day 0) and on follow-up days 7, 14, 21, and 28. PCR genotyping was performed on paired dried blood spots in the case of parasitaemia detected on or after day-7 to distinguish between recrudescence and re-infection for all treatment failures. PCR genotyping of *P. falciparum* polymorphic genes *msp1* and *msp2* was performed as per WHO protocol [29–31]. The results were classified as recrudescence if the recurrent parasites were of the same parasite strain as those on days 0 or as a new infection if they were a different strain.

### Treatment outcome classification

Treatment outcomes were classified based on parasitological and clinical outcomes as recommended by the WHO [20]. Efficacy was evaluated using microscopy and qPCR in conjunction with clinical signs and symptoms. Parasite genotyping was also performed at baseline and at the time of recurrence of parasitaemia to differentiate between recrudescence and new infection. Therapeutic responses on day 28 were classified as adequate clinical and parasitological response (ACPR), or treatment failure (TF); designated as early treatment failure (ETF), late clinical failure (LCF), or late parasitological failure (LPF). The primary outcome measure was ACPR, corrected for reinfection using PCR genotyping from the day of reemergence of parasitaemia based on per protocol method and Kaplan–Meier analysis. A secondary treatment outcome was parasite clearance during the first 3 day of follow-up and patterns of fever (i.e. temperature ≥ 37.5 °C).

### Data management

Data entry and analysis was done by using the WHO designed Excel spreadsheet [32] and SPSS version 20 for windows. Cure rate on day-28 was assessed by intention to treat (ITT) and per protocol (PP) analysis. The PP Kaplan Meir was used to analyse the primary therapeutic outcomes. Briefly, PCR-uncorrected per protocol analysis excluded participants lost to follow-up and with-drawn whilst Kaplan–Meier analysis censored last day of follow-up for such participants. PCR-corrected per protocol analysis excluded participants lost to follow-up, with-drawn, with falciparum re-infection, and undetermined PCR whilst Kaplan–Meier analysis censored last day of follow-up for those lost to follow-up as well as those withdrawn or with falciparum re-infection. Study participants with undetermined PCR were also excluded in the Kaplan–Meier analysis [20]. Secondary treatment outcomes analyzed were parasite clearance during the first three days of follow-up, patterns of fever (i.e. temperature ≥ 37.5 °C and gametocyte clearance. Chi-square and Fisher’s exact tests were used to compare proportions whilst Student’s t-test was used to compare means and *p* < 0.05 was considered significant during the analysis.

## Results

### Study participant enrollment and demographic characteristics

**Fig. 1:**
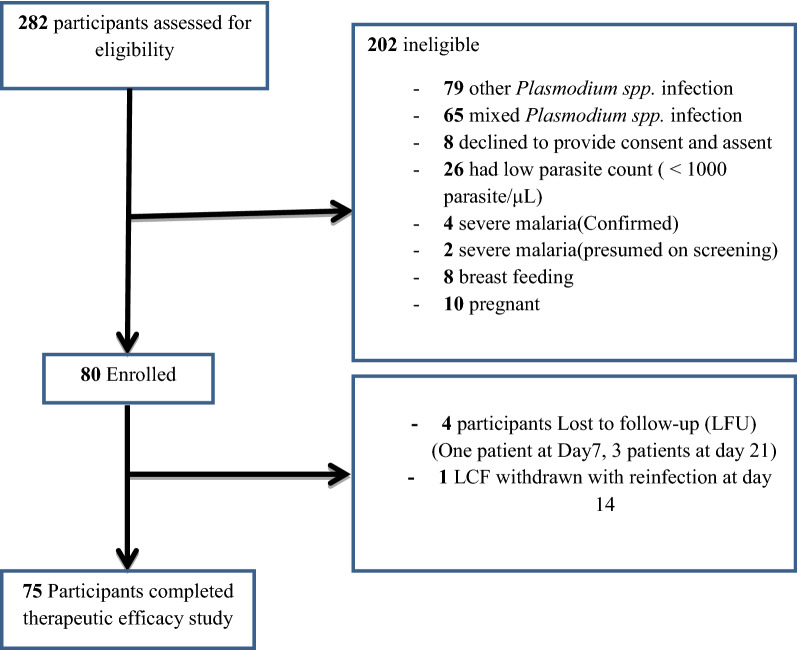
Study participant flow chart

A total of 282 febrile participants were screened and of these, 80 participants were included in this study. The rest who were not fulfilling the WHO revised protocol for malaria drug therapeutic efficacy study [20] criteria were excluded from the study. Of the 80 participants, four participants (5%) were lost to follow up on days 7 and 21 and one participant with LCF was classified as reinfection on day fourteen and was excluded from per protocol analysis. Thus, 75 participants were successfully followed up during the course of the study (Fig. 1).

The age, axillary temperature, Hb level, body weight, parasitaemia and gametocyte carriage of the study participants are summarized in Table 1. The majority (71.2%) of the study participants were males. The age group 5–15 years represented most participants 50 (62.5%) followed by the group > 15 years of age 26 (32.5%). The mean age of the study population was 20.96 (range 3–60 years). Geometric mean parasitaemia at baseline was 12,374.31 (95% CI 3699–14,744) parasite/μl among all study participants.

**Table 1:** Demographic characteristics of study participants in the evaluation of therapeutic efficacy of artemether-lumefantrine in the treatment of uncomplicated Plasmodium falciparum sssmalaria in Chewaka district, Ethiopia

Patient characteristics	Age category		
	< 15 years (n = 30)	≥15ss years (n = 50)	Total (n = 80)
Mean age ( or range)	9.27 (3–14)	27.98 (15–60)	20.96 (3–60)
Gender			
Male n (%)	37 (74.0)	20 (66.7)	57 (71.2)
Female n (%)	10 (33.3)	13 (26.0)	33 (28.8)
Temperature in ^o^C, mean (SD)	37.68 (0.58)	37.79 (0.81)	37.75 (0.73)
Weight (Kg), mean (SD)	32.77 (19.01)	63.62 (18.43)	52.05 (23.86)
Haemoglobin (g/dl), mean (SD)	11.37 (3.21)	11.82 (3.15)	11.65 (3.16)
Parasitaemia (per μl), geometric mean (range)	12,301.31 (3699–14,292)	12,418.31 (9539–14,744)	12,374.31 (3699–14,744)
Gametocyte carriage, n (%)	2 (6.7)	5 (10.0)	7 (8.8)

*SD* standard deviation, *Kg* kilogram, *Hb* haemoglobin, *n* number, *°C* degree centigrade, *g/dl* gram/decilitre

### Primary outcomes

**Fig. 2:**
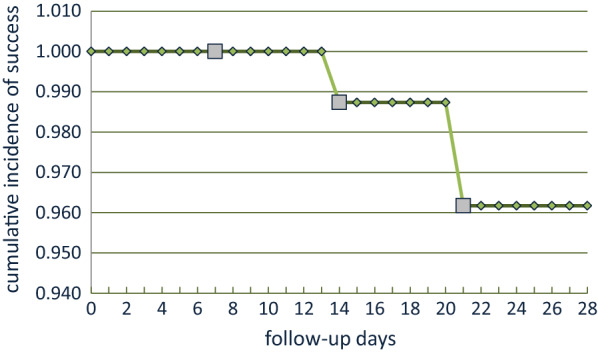
Kaplan–Meier Survival Curve with PCR corrected.  Stands for censored (lost to follow-up and re-infection)

Four treatment failures, three LPF (one case at day-14 and two cases at day-21) and one LCF case at day-14 were observed, giving PCR-uncorrected failure rate of 5.3% (4/76) (95% CI 0.8–18.2). By PCR correction, only the LCF was confirmed as a reinfection case with a failure rate of 4% (95% CI0.8–11.2). There was seen no early treatment failure (ETF). For per protocol (PP) analysis, PCR-uncorrected cure rate of AL among the study participants was 94.7% (95% CI 87.1–98.5) and the PCR corrected cure rate was 96% (95% CI 88.8–99.2). For intention to treat (ITT) analysis, the cure rate was 90% (95% CI 88.8–99.2) (Table 2). Based on PCR-corrected Kaplan–Meier survival estimate, the cumulative incidence of failure rate of AL among study participants was 3.8% (95% CI 1.3–11.4) and the cumulative incidence of success rate of AL among study participants was 96.2% (95% CI 88.6–98.7) (Fig. 2).

**Table 2:** Results of therapeutic efficacy of artemether-lumefantrine in the treatment of uncomplicated *P. falciparum* malaria in Chewaka district, Ethiopia

Treatment outcome	n (%)
ETF	0 (0.0)
LCF	1(1.3)^b^
LPF	3 (4.0)^a^
ACPR	72 (94.7)
PP PCR-uncorrected cure rate (95% CI)	72/76;94.7 (87.1–98.5)
PP PCR-corrected cure rate (95% CI)	72/75;96.0 (88.8–99.2)
ITT cure rate (95% CI)	72/80;90.0 (83.3–96.7)

*ETF* Early treatment failure, *LCF* Late clinical failure, *LPF* Late parasitological failure, *ACPR* Adequate clinical and parasitological response, *WTH* Withdrawal, *LFU* Lost to follow-up, *n* number, *PP* per protocol analysis, *ITT* intention to treat analysis, *LFU* loss to follow up

^a^Stands for recrudescence and ^b^Stands for new infection

### Secondary outcomes

#### Parasite clearance

Based on qPCR quantitative parasite assessment, parasitaemia detection rate was 38.8% (31/80) on day-1, and declined to 18.8% (15/80) on day-2 and 3.8% (3/80) on day-3. Accordingly, parasite clearance rate was high that 61.2% of the participants cleared parasitaemia on day-1, 81.2% on day-2 and 96.2% on day-3 (Table 3). In total, only three participants (3.8%) of the 80 study participants were found to be positive on day-3. The day-3 positive participants were followed up to day 28 and had ACPR. Parasite clearance rate was compared between age groups taking day-2 mean parasitaemia as comparison variable; day-2 mean parasitaemia was significantly higher (*p* < 0.05) in age group less than 15 years (702.4 ± 1390.46) compared to age group greater than 15 years (408.3 ± 1041.09) implying that study participants age greater than 15 years cleared the parasite faster than study participants age less than 15 years.

**Table 3:** Parasite, fever and gametocyte clearance rate in study participants during supervised treatment and follow-up period in Chewaka district, Ethiopia

Variable	Age category	Follow-up days			
		D0 (Baseline)	D1 (24 hr)	D2 (48 hr)	D3 (72 hr)
Parasitaemia detected, n (%)	< 15 yrs	30 (100)	12 (40.0)	9 (30.0)	0 (0.0)
	≥ 15 yrs	50 (100)	19 (38.0)	7 (14.0)	3 (6.0)
	Total	80 (100)	31(38.8)	16 (20.0)	3 (3.8)
Fever cases (≥ 37.5 °C), n (%)	< 15 yrs	24 (80)	17 (56.7)	7 (23.3)	0 (0.0)
	≥ 15 yrs	35 (70)	21 (42.0)	4 (8.0)	2 (6.7)
	Total	59 (73.8)	38 (47.5)	11 (13.8)	2 (2.5)
Gametocytes carriage, n %)	< 15 yrs	2 (6.7)	0 (0.0)	0 (0.0)	0 (0.0)
	≥ 15 yrs	5 (10.0)	4 (8.0)	3 (6.0)	0 (0.0)
	Total	7 (8.8)	4 (5.0)	3 (3.8)	0 (0.0)

*D0* Day 0, *D1* Day 1, *D2* Day 2, *D3* Day 3, *°C* degree centigrade, *n* number of participants, *yrs* years, *hr* hour

#### Fever clearance

**Fig. 3:**
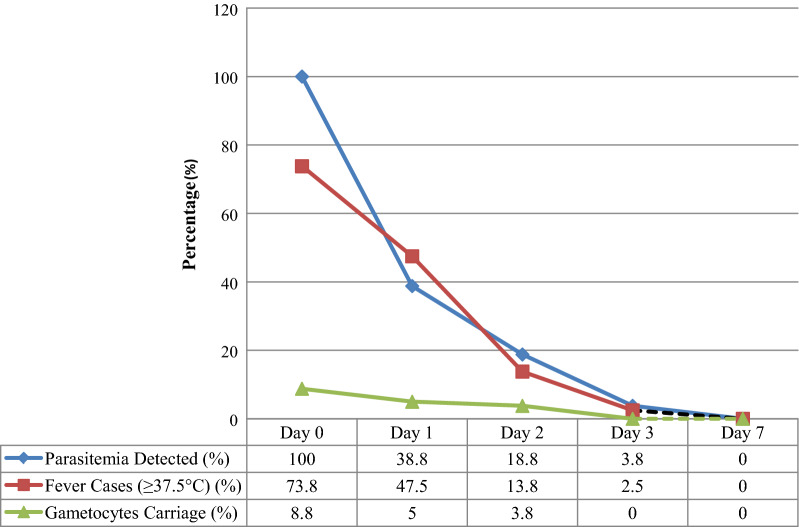
Parasitemia, fever and gametocyte clearance rate during treatment and follow-up period in Chewaka district, Buno Bedele Zone, Ethiopia

Febrile individuals, with ≥ 37.5 °C axillary temperature, accounted for 73.8% (59/80) at the day of recruitment and decreased to 47.8% (38/80) on day 1, 13.8% (11/80) on day-2 and 2.5% (2/80) on day-3. Accordingly, fever clearance rate was 87.2% (69/80) on day-2, 97.5% (74/80) on day-3 (Table 3 and Fig. 3) and no febrile case was detected onwards except the three recrudescence and the one reinfection cases.

#### Gametocytaemia clearance

Based on microscopic analysis, only 8.8% (7/80) gametocyte carriers, among all study participants, were detected at enrolment: Of these, 6.2% (2/30) detected in < 15 years and 10% (5/50) was detected in ≥ 15 years. Of the day-0 gametocyte carriers detected, three cases on day-1 and four cases on day-2 were cleared giving gametocytaemia clearance rate of 42.9% (3/7) and/or 57.1% (4/7) respectively. The proportion of gametocyte carriage per total study participants was declined from 8.8% (7/80) on day 0 to 5% (4/80) on day 1, 3.8% (3/80) on day 2 and totally disappeared on day 3 (Table 3 and Fig. 3). After initiation of the treatment no new gametocyte carrier was observed.

### Clinical cases with recrudescence

Three study participants experiencing recrudescent parasitaemia between 14 and 21 days after treatment start. All attended their treatment at health centre for uncomplicated *P. falciparum* malaria and had an adequate initial treatment response and were parasite-negative by microscopy and qPCR analysis latest on the second day after initiated AL treatment of admission. Each dose was administered by a ward nurse and medical officer who documented intake in the medical record, and compliance according to documentation was 100%. The participants were 21–51 years old with body weight 54–70 kg.

### Adverse drug reactions

Information on AL related side effects was collected through self-reporting and recorded in the case reporting forms. No serious adverse events were reported throughout the 28 days follow up period. Overall, 80% of all study participants reported no side effects and 20% reported one or two side effects. The most reported adverse reactions in this study were headache (8.8%), vomiting (3.4%), Shortness of breath (2.5%), cough (1.3%), diarrhea (1.3%) and joint/muscle pain (2.5%). Most of these probable AEs disappeared with the clearance of parasitaemia except cough. Cough persisted for some time beyond parasite clearance.

## Discussion

In this study, the PCR-corrected cure rate 96% (95% CI 88.8–99.2), which showed the high therapeutic efficacy of AL since its introduction for the treatment of uncomplicated falciparum malaria in the study area, meeting the WHO recommendation that cure rates for falciparum malaria should be at least 90% [20]. The observed PCR corrected cure rate in this study is comparable with what was documented in other parts of Ethiopia by Nega et al. Mekonnen et al. and Getnet et al. in which PCR-corrected ACPR of 97.8, 98.8 and 95% respectively, were observed after 28 days follow following treatment with AL [22–24]. The observed high AL cure rate is comparable with other findings in other parts of Africa in which PCR-corrected ACPR of 95% in Ghana by Abuaku et al*.* and 99.3% in Tanzania by Ishengoma et al. were demonstrated [33, 34]. Most of these results are well within the confidence intervals of this study and minor differences may be attributable to host nutritional and immune status, initial parasitaemia level, pharmacokinetics and pharmacodynamics may influence the therapeutic efficacy of a drug apart from inherent parasite susceptibility [35].

Early treatment failure was not observed in this study, whereas three LPF were 3.9% (95% CI 0.8–11.1) in PCR uncorrected and 4% (95% CI 0.8–11.2) treatment failure was observed in PCR corrected data. Several studies [36–39] in which therapeutic efficacy tests were combined with sampling of plasma or whole blood for drug concentration measurements at various times during follow-up have shown that cured patients have higher drug concentrations than those in whom treatment failed. There are two possible explanations for the latter finding. First, failures are associated with inadequate drug concentrations rather than resistance-this could be the case in our findings of treatment failure of 4%; secondly, when drug resistance emerges, there is a higher likelihood that a resistant strain will emerge if the drug is present at a suboptimal concentration.

According to the Kaplan Meier PP survival analysis in the current study, the PCR-corrected AL failure rate was 3.8% (95% CI 1.3–11.4) and the cumulative incidence of success rate of AL among study participants was 96.2% (95% CI 88.6–98.7) (Fig. 2). The AL treatment failure rates observed is below the WHO threshold of 10%, and, therefore, suggests that lumefantrine was not failing as partner drug in AL use in the study area, with no change in the national treatment policy [20]. Inadequate drug absorption resulting in suboptimal serum drug concentrations can cause treatment failure. Artemether is rapidly absorbed and eliminated (half-life of a few hours), whereas lumefantrine was variably absorbed and more slowly eliminated [12]. Lumefantrine is a lipophilic compound with erratic bioavailability unless administered with a small fatty meal [40], and for this reason, guidelines recommend administration of AL with a fatty meal such as milk or a small biscuit. In the case of our study, we were unable to confirm adequate serum concentrations of lumefantrine; however, all participants received a complete course of treatment and all the doses were supervised in the health Centre and administered with a milk biscuit to ensure good absorption. Nevertheless, considerable inter-individual variation exists in lumefantrine exposure, and these participants may have had relatively low concentrations.

Day-3 parasitaemia may be a poor predictor of patient outcomes on day 28 because the supplemental drug may still clear the infection. However, determining the presence of day 3 parasitaemia has been suggested as a surrogate for assessing artemisinin resistance e.g. in mobile populations [41]. In the present study, the parasitaemia on day 3 following treatment with AL was only 3.8% (95% CI 0.8–10.6) on day-3 and Day-3 parasitaemia did not correspond to failures observed during follow-up. This may indicate absence of resistant strains of P. falciparum to artemisinin in study area. This is in line with the WHO 2009 anti-malarial protocol, that if 10% of the study participants have peripheral parasitaemia on day 3, it is an indicator of emergence of artemisinin resistance to Plasmodium species [20, 42]. The overall rate of day-3 positivity observed in this study are consistent with the 3–5% background rate of day-3 positivity that might be expected in the absence of resistance to artemisinin, but also the 3–10% range which in the past has been seen as appropriate window for initiating containment activities [43].

Episodes of recurrent parasitaemia following treatment may be due to recrudescence of the initial infection, reflecting failure of the drug to clear the infection; or, they may be due to new infections that occurred during the follow-up period [44]. In areas of high endemicity recurrent infections are common although PCR analysis of *msp1* and *msp2* gene markers estimated that three cases were recrudescence and a single case of re-infections are observed in this study. The recrudescent parasitaemia resolved quickly after initiated re-treatment in all cases.

AL showed rapid parasite and fever clearance during the first 3 days of controlled supervised follow-up period. Over all prevalence of parasite and fever declined by 96.2 and 97.5% on day 3 respectively. Gametocytaemia was absent on day 3 following treatment with AL. These findings suggest that AL remains effective in rapidly clearing asexual parasites and fever as well as reducing gametocyte carriage rates in study Ethiopia [22–24]. The high parasite and fever clearance rates could be explained by the fast act of artemether to clear parasite biomass leading to rapid resolution of clinical manifestations [12, 45].

This study also showed that AL had a safety profile comparable to previous studies and was well tolerated with minimal adverse events. Studies conducted in other African countries [46–48] reported similar safety profiles of AL when used for the treatment of uncomplicated falciparum malaria. A high number of cases reporting cough at the study site could be attributed to weather conditions, which were relatively cold and rainy at the time of the study.

The relative bioavailability of artemether and lumefantrine increases by 2–3 times and 16 times, respectively, when administered after a high-fat meal [49]. The limitation of this study is the lack of pharmacokinetic data to better explain the recrudescence observed. The cure rates for AL may therefore be higher than the rates observed in this study.

## Conclusions

The findings of this study showed that the therapeutic efficacy of AL is considerably high (above 90%). AL remains highly efficacious in the treatment of uncomplicated malaria in the study area achieving rapid fever and parasite clearance as well as low gametocyte carriage rates despite the use of this combination for more than 15 years. Day-three parasitaemia warrants a close monitoring of the efficacy of AL in the future and should be continued in order to generate evidence to support national malaria treatment policy and practice.

## Data Availability

Springer Nature remains neutral with regard to jurisdictional claims in published maps and institutional affiliations.
